# Targeting Precision in Cancer Immunotherapy: Naturally-Occurring Antigen-Specific TCR Discovery with Single-Cell Sequencing

**DOI:** 10.3390/cancers16234020

**Published:** 2024-11-30

**Authors:** Saleh Alrhmoun, Marina Fisher, Julia Lopatnikova, Olga Perik-Zavodskaia, Marina Volynets, Roman Perik-Zavodskii, Julia Shevchenko, Kirill Nazarov, Julia Philippova, Alaa Alsalloum, Vasily Kurilin, Alexander Silkov, Sergey Sennikov

**Affiliations:** 1 Laboratory of Molecular Immunology, Research Institute of Fundamental and Clinical Immunology, 630099 Novosibirsk, Russia; msolshanova@gmail.com (M.F.); lopatnikova18@yandex.ru (J.L.); perik.zavodskaia@gmail.com (O.P.-Z.); mrsmarinavolynets@gmail.com (M.V.); zavodskii.1448@gmail.com (R.P.-Z.); airyuka@mail.ru (J.P.);; 2 Department of Natural Sciences, Novosibirsk State University, 630090 Novosibirsk, Russia; 3 Institute of Medicine and Medical Technologies, Department of Immunology, Novosibirsk State University, 630090 Novosibirsk, Russia

**Keywords:** HER2/neu, single-cell sequencing, TCR-T cell therapy, adoptive cell therapy, cancer immunotherapy, naturally occurring TCRs

## Abstract

**Background**: Adoptive cell therapy is the most promising approach for battling cancer, with T cell receptor-engineered T (TCR-T) cell therapy emerging as the most viable option for treating solid tumors. Current techniques for preparing TCR-T cell therapy provide a limited number of candidates TCRs, missing the comprehensive view of the repertoire, which may hinder the identification of the most effective TCRs. **Methods**: Dendritic cells were primed with immunogenic peptides of the antigen of interest to expand antigen-specific CD8 T lymphocytes from peripheral blood. Following that, the entire repertoire of naturally occurring antigen-specific TCRs was analyzed using single-cell RNA sequencing, alongside the assessment of the dominancy, transcriptome, and binding specificity of the obtained clonotypes, utilizing the TCRscape tool and ERGO-II neural network to identify the most effective candidate for TCR-T cell therapy development. Finally, TCR-T cells with the candidate TCR were obtained, followed by assessing their functionality and selectivity. **Results**: The developed protocol achieved a remarkable increase in the percentage of antigen-specific T cells by more than 200-fold, with more than 100 antigen-specific TCR clonotypes identified. The resulting TCR-T cells demonstrated high cytotoxicity and selectivity for the targeted antigen, indicating their potential to preferentially target tumor cells. **Conclusions**: This study offers a comprehensive approach for the discovery and analysis of not only few, but the entire repertoire of naturally occurring antigen-specific TCRs for TCR-T cell therapy development. Additionally, the proposed approach can be tailored to accommodate different types of antigens and MHC variants, making it a highly versatile tool for both research and clinical applications.

## Simple Summary

T cell receptor-engineered T (TCR-T) cell therapy, which engineers the cells of the immune system to enhance their ability for targeting tumor cells, holds great promise, especially for solid tumors. One major challenge in this field is the identification of the right T Cell Receptor, which constitutes the core of developing these therapies. Our study introduces a new approach for the discovery and analysis of the entire repertoire of naturally-occurring antigen-specific TCRs utilizing state-of-the-art single-cell sequencing technology. This method could greatly improve the development of personalized cancer therapies and may lead to more effective treatments for a variety of tumors.

## 1. Introduction

Cancer remains a widespread, predominant, and life-threatening global health challenge, with the number of new cases increasing every year [1]. Each year, approximately, 19–20 million people are diagnosed with cancer worldwide, and over fifty percent of cancer patients face mortality [2]. Notably, solid tumors contribute significantly to the overall burden of cancer cases and annual cancer-related deaths globally [3].

Clinical immunotherapy has advanced significantly in recent decades, with the development of immune checkpoint inhibitors and Chimeric Antigen Receptor T cell (CAR-T) therapy. Nonetheless, their efficacy is limited in the majority of solid tumors, emphasizing the need to investigate innovative immunotherapeutic approaches [4,5]. In addressing this issue, a compelling solution lies in leveraging the immune system’s weaponry against tumors, particularly by harnessing the primary adaptive immune effector cells: T lymphocytes and their specialized T-cell receptors (TCRs). T cell receptor-engineered T (TCR-T) cell therapy emerges as a viable alternative, with numerous major advantages [5,6]. Firstly, TCR-T cells can target more antigens compared to CAR-T cells, as they can recognize epitopes derived from both cell surface and intracellular proteins, presented by the major histocompatibility complex (MHC), whereas CAR-T cells are limited to cell surface antigens. This capability allows TCR-T cells to target approximately 88% of proteins that reside exclusively within intracellular compartments [7]. Secondly, TCR-T cells require fewer epitopes for activation compared to CAR-T cells (1–3 vs. 10^3^ epitopes per cell) [8]. This increased sensitivity may enhance the detection and elimination of tumor cells. At the same time, TCRs exhibit remarkable specificity, as they can discriminate between peptides that differ by a single amino acid. Nevertheless, a notable disadvantage of TCR-T cell therapy is its restriction by MHC molecules. This restriction implies that any specific TCR can only be used to treat patients who have a compatible MHC phenotype [5,6,7].

The Human Epidermal Growth Factor Receptor 2 (HER2), also known as CD340 or p185, is a member of the epidermal growth factor family of tyrosine kinase receptors (EGFRs/ErbBs), which consists of four members: HER1 (EGFR, erbB1), HER2 (HER2/neu, EGFR-2, erbB2), HER3 (EGFR-3, erbB3), and HER4 (erbB4). HER2/neu play a pivotal role in the pathogenesis and progression of numerous cancers. The significance of this receptor stems from its overexpression across multiple cancer types, with this overexpression often associated with more aggressive disease, higher recurrence rates, and shorter survival periods [9]. These findings imply a substantive involvement of HER2/neu receptor overexpression in the pathogenesis of these cancers. On top of that, this increased expression can result in a 100–200-fold increase in the concentration of the HER2/neu receptor within tumor tissues compared to normal tissue levels, highlighting the potential role for HER2/neu as a prominent tumor-associated antigen (TAA) [9,10].

In this article, we demonstrate the application of state-of-art Single-Cell Sequencing technology, the TCRscape tool (https://github.com/Perik-Zavodskii/TCRscape, accessed on 1 May 2024) [11], and the powerful capabilities of machine learning algorithms via the use of ERGO-II (https://github.com/IdoSpringer/ERGO-II, accessed on 1 May 2024) [12] for the development of a protocol to analyze the entire repertoire of naturally-occurring TCRs specific to the tumor-associated antigen HER2/neu and identifying candidate TCRs potentially suitable for designing TCR-T adoptive cell therapy.

## 2. Materials and Methods

### 2.1. Study Population

The study population consisted of seven conditionally healthy adult donors who had been pre-selected for the presence of the HLA-A02 allele based on the results of a flow cytometry screen using PE antihuman HLA-A2 antibodies (343306, Biolegend, San Diego, CA, USA). The average age of the donors was 27.33 ± 6.34 years (mean ± SE). Among them, four donors were females, and three were males. All the donors signed an informed consent to participate in the study.

### 2.2. PBMCs Isolation

Peripheral blood was collected into vacuum tubes with EDTA anticoagulant, and the peripheral blood mononuclear cells (PBMCs) were isolated using a conventional Ficoll–Urografin density gradient method. Additional details can be found in the Supplementary Materials.

### 2.3. Clonal Expansion of HER2/Neu-Specific CD8+ Lymphocytes

The HER2/neu HLA-A2-binding peptide KIFGSLAFL (KIF, p369-377) was synthesized by Immunotex (Stavropol, Russia).

MHC tetramers (HLA-A*02:01) loaded with the HER2/neu peptide KIF and labeled with either phycoerythrin (PE) or allophycocyanin (APC) fluorophores were obtained using the Flex-T technology (280004, BioLegend, San Diego, CA, USA) according to the manufacturer’s instructions. The efficiency of peptide exchange was qualitatively evaluated using the LEGEND MAX™ Flex-T™ Human Class I Peptide Exchange ELISA Kit (447207, BioLegend, San Diego, CA, USA) according to the manufacturer’s instructions. The results showed 80% efficiency in replacing the UV light labile peptide. The obtained Flex-T tetramers were then utilized to evaluate the content of KIF-specific T cells among lymphocytes.

To induce preparative amounts of antigen-specific cytotoxic T lymphocytes in vitro, DCs were utilized, as they have a unique ability to capture antigens, followed by their processing and presentation in combination with MHC class I and class II molecules for the activation of naive T cells, leading to their clonal expansion and differentiation into effector cells [13]. Based on a well-established in-lab protocol that has proven its effectiveness in previous work [14,15,16], a multi-stage, maximally optimized and efficient protocol has been developed. All the reported concentrations are given in the form of final concentrations:

#### 2.3.1. Stage 1—Obtaining Mature DCs

PBMCs, isolated from the blood of HLA-A02+ conditionally healthy donors, were separated into the corresponding adhesive and non-adhesive fractions based on their ability to adhere to plastic surfaces after a 30 min incubation period at 37 °C in the CO_2_ incubator. To obtain immature DCs, the adhesive fraction of PBMCs (20–25 million cells, 1–2 million cells/mL) was cultured into 75 cm^2^ tissue culture flasks (TPP, 90075, Trasadingen, Switzerland) in the presence of recombinant human granulocyte-macrophage colony-stimulating factor 100 ng/mL (GM-CSF, 572904, BioLegend, San Diego, CA, USA) and interleukin-4 50 ng/mL (IL-4, 574006, BioLegend, San Diego, CA, USA) for 4 days, with partial media replacement on the 3rd day of cultivation. On the 5th day, the peptide of interest was introduced into the culture at a concentration of 30 μg/mL, followed by the induction of DC maturation using TNF-alpha 25 ng/mL (TNF-a, 570108, BioLegend, San Diego, CA, USA) on the 6th day of cultivation.

#### 2.3.2. Stage 2—Obtaining CD8+ T Cells

Simultaneously, the non-adhesive fraction of PBMCs was utilized for the isolation of CD8+ T cells using MojoSort negative magnetic selection Human CD8 T Cell Isolation Kit (480012, BioLegend, San Diego, CA, USA) according to the manufacturer’s instructions. Immediately after the isolation, CD8+ T cells (2 million cells/mL) were stimulated with interleukin-7 (IL-7, 581906, BioLegend, San Diego, CA, USA), interleukin-15 (IL-15, 570306, BioLegend, San Diego, CA, USA), and interleukin-2 (IL-2, 589106, BioLegend, San Diego, CA, USA) at a concentration of 10 ng/mL each for 6 days, with total media replacement on the 3rd day of cultivation and the addition of a fresh portion of IL-2/7/15, alongside anti-CD3 0.5 μg/mL (830301, BioLegend, San Diego, CA, USA) and anti-CD28 1 μg/mL (302902, BioLegend, San Diego, CA, USA). On the 6th day of cultivation, the conditioned media of this culture were reserved for later use.

#### 2.3.3. Stage 3—Co-Cultivation

Mature DCs after antigen loading, at a concentration of 1–2 million/mL, were co-cultured with stimulated CD8+ T cells in a new 75 cm^2^ tissue culture flask on the 7th day from the start of the experiment, at a ratio of 1:10 (DCs:CD8+). The first 3 days of co-cultivation were carried out without additional stimulation to selectively eliminate cells that were not receiving stimulation through their T-cell receptor from DCs, but on the 4th day, the co-culture media were supplemented with anti-CD3 0.5 μg/mL, anti-CD28 1 μg/mL, IL-2, IL-7, and IL-15 each at a concentration of 10 ng/mL to maintain viability and promote proliferation of the obtained CD8+ cytotoxic T lymphocytes.

#### 2.3.4. Stage 4—Isolating Antigen-Specific T Cells

After 7–8 days of co-culturing DCs and CD8+ T cells, antigen-specific T cells were isolated from the co-culture using a two-step isolation process. The DCs were first eliminated using the MojoSort negative magnetic selection Human CD8 T Cell Isolation Kit (BioLegend, San Diego, USA), and then the antigen-specific T cells were isolated using Flex-T tetramers loaded with the corresponding peptide, having positive cells stained simultaneously with two fluorochromes for their identification and sorting on a BD FACS Aria I sorter (BD Biosciences, San Jose, CA, USA) (Figure S2) with the following sorting parameters: pressure of 20 psi, mode set to “Purity”, speed of up to 2500 events/s. Briefly, morphologically viable lymphocytes were gated from the forward and side scatters gate, after which the antigen-specific T cell population was gated based on the fact that it should be stained simultaneously by tetramers coupled to either APC or PE fluorochromes (double-positive cells).

#### 2.3.5. Stage 5—Stimulation of Cell Proliferation

Following sorting, the antigen-specific T cells were transferred to a flat-bottomed culture plate at a concentration of 2–4 million/mL, where they were cultured under constant supervision in the presence of the following stimulants: anti-CD3 0.5 μg/mL, anti-CD28 1 μg/mL, IL-2, IL-7, and IL-15 each at a concentration of 10 ng/mL. The culture media consisted of equal parts of RPMI-1640 (1.3.4., Biolot, St. Petersburg, Russia) and the conditioned media from the CD8+ T cells culture. When assessing the state of cells, special attention was paid to the formation of cytotoxic lymphocyte aggregates within the culture, and the cells were reseeded, as needed, to achieve the desired number of cells for downstream application. The total duration of cultivation of antigen-specific T cells ranged from 14 to 21 days. The enrichment results were visualized in GraphPad Prism 10.2.3 using bar plots.

### 2.4. Sample Tag Sample Barcoding and Cell Counting for BD Rhapsody Single-Cell Analysis

After preparing the HER2/neu-specific CD8+ T lymphocytes, we incubated cells from different donors (*n* = 3, the other 4 donors were excluded due to the low percentage of antigen-specific T-cells) with Sample Tag antibodies from the BD™ Single-Cell Multiplexing Kit (633781, BD Biosciences, San Jose, CA, USA) for 20 min at room temperature. After three washing cycles, cells were stained with Calcein (564061, BD Biosciences, San Jose, CA, USA) according to the BD Rhapsody Single-Cell Analysis System User Guide instructions, counted using the Attune NxT flow cytometer (Thermo Fisher, Waltham, MA, USA) and the automated cell counter Countess 3 (Thermo Fisher, Waltham, MA, USA), pooled together, and resuspended in cold sample buffer of BD Rhapsody to a final concentration of 40 cells/µL and a volume of 620 µL for loading into BD Rhapsody Cartridge. The quality and quantity of cell loading into the cartridge was visually assessed using IN Cell Analyzer 6000 (GE HealthCare, Chicago, IL, USA) with the help of Calcein.

### 2.5. cDNA Library Preparation and Sequencing

We performed single-cell capture and library preparation using the BD Rhapsody Express Single-Cell Analysis System (BD Biosciences, San Jose, CA, USA), according to the manufacturer’s instructions (TCR/BCR Full Length, Targeted mRNA, and Sample Tag Library Preparation Protocol), using BD RhapsodyTM Targeted mRNA and AbSeq Amplification Kit (633774, BD Biosciences, San Jose, CA, USA) and BD RhapsodyTM TCR/BCR Amplification Kit (665345, BD Biosciences, San Jose, CA, USA). Briefly, we captured single cells in the BD Rhapsody cartridge, added magnetic beads for poly-A based mRNA and Sample Tag capture, lysed the cells, performed reverse transcription of the poly-A captured mRNA and Sample Tag molecules on the magnetic beads, added template switch oligos, performed another round of reverse transcription, denaturated the Sample Tag cDNA, performed Sample Tag PCR1, performed Klenow DNA polymerase fragment-based extension of the cDNA on the beads, treated the beads with Exonuclease I, amplified the cDNA using the TCR primers, denaturated and collected the TCR PCR1 amplicons, and performed another round of PCR using Human Immune Response Primer Panel (mRNA panel for short) (633750, BD Biosciences, San Jose, CA, USA)—containing 399 primer pairs targeting 397 different gene. After that we collected the mRNA panel PCR1 amplicons and then purified the resulting PCR1 products (TCR, mRNA panel and Sample Tag products) on the basis of the amplicon size using AMPure XP magnetic beads (A63880, Beckman Coulter, Brea, CA, USA). After that, we further amplified the purified TCR, mRNA panel, and Sample Tag PCR1 products, and we purified the resulting PCR2 products according to size selection using the AMPure XP magnetic beads. As for the TCR PCR2 amplicons, we normalized their concentration to 1.8 ng/μL, performed random primer extension (TCR RPE) using random primers and Klenow DNA polymerase fragment, and then performed TCR RPE library clean-up using double-sided selection with the help of AMPure XP magnetic beads. Subsequently, we assessed the concentration of the mRNA panel and Sample Tag PCR2 and TCR RPE products by Qubit 4 and the Qubit dsDNA High-Sensitivity Assay Kit (Q32854, Thermo Fisher, Waltham, MA, USA). From there, we normalized the concentration of the PCR2 products to 1.0 ng/μL for the mRNA panel and 1.0 ng/μL for the Sample Tag; as for the TCR, the RPE product was used undiluted. Eventually, we performed a final round of amplification using indexes for Illumina sequencer to prepare the final libraries. We quantified the final libraries using Qubit 4 and Agilent BioAnalyzer 2100 (Agilent, Santa Clara, CA, USA) and pooled them with the following ration (~81/12/7% TCR/mRNA/Sample Tag, estimated 15000 read/cell for TCR, 2200 read/cell for mRNA panel and 1200 read/cell for Sample Tag) to achieve a final concentration of 2 nM. The final pooled libraries were sequenced (150 bp paired-end, 600 million clusters) on a NovaSeq 6000 sequencer (Illumina, San Diego, CA, USA).

### 2.6. Sequencing Data Processing

We processed the FASTQ files obtained from sequencer using the BD Rhapsody pipeline 1.11.1L (BD Biosciences, San Jose, CA, USA). The pipeline removed read pairs with low quality based on their read length, mean base quality score, and highest single-nucleotide frequency; analyzed the remaining high-quality R1 reads to identify cell label and unique molecular identifier (UMI) sequences; aligned the remaining high-quality R2 reads to the reference panel sequences (mRNA) using Bowtie2; collapsed reads with the same cell label, the same UMI sequence, and the same gene into a single molecule; adjusted the obtained counts by error correction algorithms, namely, recursive substitution error correction (RSEC) and distribution-based error correction (DBEC), in order to count for sequencing and PCR errors; and estimated cell counts using the second derivative analysis to filter out noisy cell labels by looking for the one inflection point and considering cell labels after that point to be noisy labels. Then, the pipeline used the molecular barcoded oligo-conjugated antibodies (BD™ Single-Cell Multiplexing Kit) to demultiplex the samples and filter out the multiples, which resulted in the pipeline calling 4989 single cells. Following that, the pipeline performed the per-cell alignment of TCR library reads to create TCR contigs, annotated the contigs, and created gene expression matrices for each biological sample and a cumulative Adaptive Immune Receptor Repertoire matrix of the TCR contigs.

### 2.7. ERGO-II TCR-Peptide-MHC Binding Specificity Prediction and TCRscape-Visualize Clonotype Selection

We downloaded the ERGO-II neural network [17] and launched the tool from the terminal with the selection of the input file and database (McPAS-TCR) [18]. McPAS-TCR is a manually curated database based on published literature containing information on forty thousand T cell receptor sequences and the antigens they recognize, including T cell type (CD4/CD8) and MHC type (MHC-I/MHC-II class). McPAS-TCR includes information about T lymphocytes that span various human or mouse pathological conditions (including viral infections, cancer, and autoimmune reactions). ERGO-II requires information about TCR CDR3α and CDR3β sequences, peptide sequence, MHC type, V and J genes, and T cell type. The output file contained a prediction value for the binding of T-cell receptor to the peptide/MHC complex, which ranged from 0 to 1, where 0 is the minimum predicted binding score, and 1 is the maximum predicted binding score.

**Figure 1:**
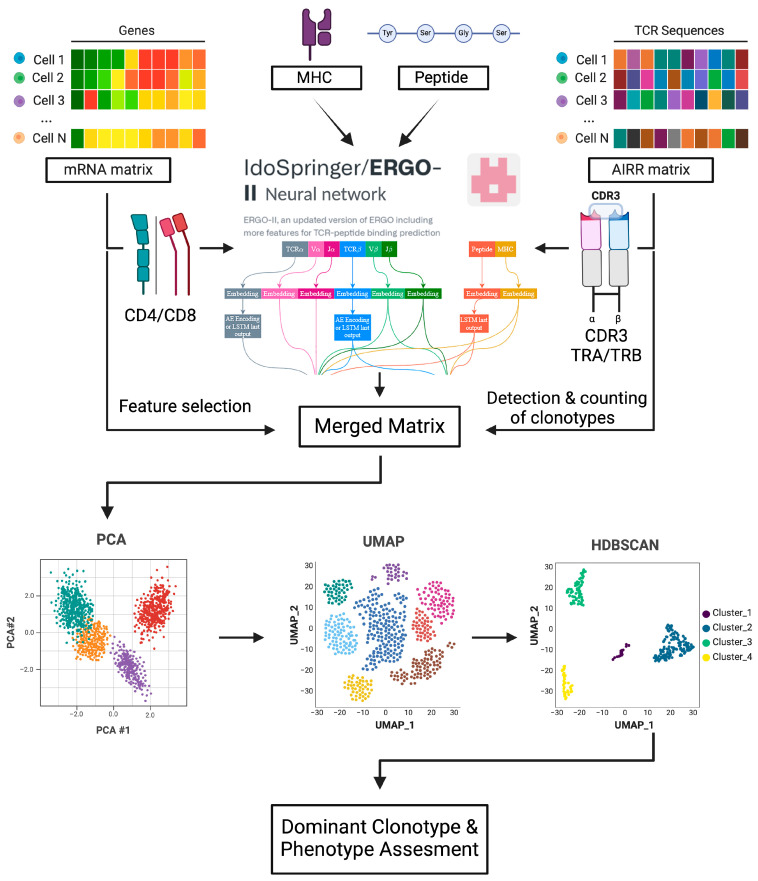
Overview of the sequential steps in the TCRscape tool, created with BioRender.com.

We then imported the gene expression matrices of each biological sample and predicted TCR–peptide–MHC binding scores and the Adaptive Immune Receptor Repertoire (AIRR) matrix into TCRscape [11] (Figure 1); this was followed by performing log2 (Counts Per Million CPM) normalization on the merged matrix and counting full-length TCR clonotypes; we then performed Principal Component Analysis (PCA) for the merged matrix to assess data dimensionality and Uniform Manifold Approximation and Projection (UMAP) dimensionality reduction to co-embed the clonotype and predicted binding score and gene expression data. After that, we clustered the cells using HDBSCAN [19] and found the dominant clonotype using the predicted binding score and cell number per clonotype. Finally, we exported the dominant clonotype data.

### 2.8. Transfer Plasmid Construction

The sequences of TCRa and TCRb alongside the other elements of the insert were optimized for insertion ptimization Tool (https://www.novoprolabs.com/tools/codon-optimization, accessed on 1 June 2023) [20] with the help of BamHI and BsrGI restriction sites. The plasmid was synthesized by Lumiprobe RUS Ltd. (Moscow, Russia). The transfer plasmid was designed based on the HIV-1 pLenti hPGK GFP vector (Figure S3) with an insert replacing the EGFP gene. This vector was used to obtain Lentiviruses carrying the gene encoding the chosen TCR clonotype that specifically targets the HER2/neu antigen.

### 2.9. Lentivirus Preparation

The transfer plasmid, VSV-G encoding plasmid, and third generation lentivirus packaging plasmids Gag-Pol and Rev (Life Science Market, Beijing, China) were amplified in *E. coli*, NEB Stable strain (C3040I, New England Biolabs, Ipswich, MA, USA), and isolated using the Maxi-prep kit for isolation of plasmid DNA from bacterial cells (Plasmid-20 maxi, Biolabmix, Moscow, Russia) according to the manufacturer’s instructions, the isolated plasmids were verified using restriction enzymes and gel electrophoresis.

The Human embryonic kidney 293T (HEK293T) packaging cells, generously provided by Dr. Hiroshi Shiku (Mie University, Tsu, Mie Prefecture, Japan), were used for the production of lentiviruses. The transfection was carried out using Lipofectamine 2000 (11668500, Thermo Fisher, Waltham, MA, USA) according to the manufacturer’s instructions. Following 72 h after transfection, the supernatant containing lentivirus was collected and concentrated using TransLv™ Lentivirus Precipitation Solution (5×) (FV101-01, TransGen, Beijing, China) according to the manufacturer’s instructions. Finally, the concentrated lentivirus solution was transferred to a cryoprobe that was kept at −150 °C until later use. Additional details can be found in the Supplementary Materials.

### 2.10. Lentivirus Titration

For titration of the obtained lentiviruses, the TransLv™ Lentivirus qPCR Titration Kit (FV201-01, TransGen, Beijing, China) was used according to the manufacturer’s instructions. HEK293T cells were selected as target cells due to their high susceptibility to transduction. Additional details can be found in the Supplementary Materials.

### 2.11. HER2/Neu-Specific TCR T-Cell Manufacturing

On day 1, in preparation for the transduction, Retronectin 5 µg/mL (Sci Store, Moscow, Russia) and anti-CD3 antibodies in an amount of 5 µg/mL in Acid Citrate Dextrose Solution A (ACDA) were adsorbed to the wells of a 24-well plate at 415 µL/well overnight. After that, on day 2, PBMCs were isolated from the peripheral blood of conditionally healthy donors on Ficoll gradient followed by CD3+ cells isolation using MojoSort negative magnetic selection with the Human CD3 T Cell Isolation Kit (480022, Biolegend, San Diego, CA, USA) according to the manufacturer’s instructions. The CD3+ cells at a dose of 7.5 × 10^5^ cells/mL in RPMI-1640 medium were then incubated in the plate with the retronectin and anti-CD3 antibodies in addition to 300 units/mL of IL-2 for 48 h (2 mL/well).

On day 3, Retronectin 5 µg/mL in ACDA was applied to the wells of a 24-well plate at 255 µL/well overnight. After that, on day 4, CD3+ cells were collected from the plate, centrifuged at 350 g for 10 min, and resuspended at a concentration of 4 × 10^5^ cells/mL in GT-T551 (WK552S, Takara Bio, Mountain View, CA, USA) serum-free medium. Next, 500 µL of cell suspension (2 × 10^5^ cell) was added to each well of the 24-well plate with Retronectin, followed by adding the lentivirus, with protamine sulfate 5–8 µg/mL (Ellara, Moscow, Russia) at an MOI of 1 that was optimized beforehand for CD3+ cells. The plate was then centrifuged for 2 h at 600 g and 32 °C. After the centrifugation, 500 µL of warm GT-T551 with IL-2 to a final concentration of 300 units/mL (in the well) were added and the cells were then allowed to incubate overnight.

The following step, on day 5, was to transfer the cells to the wells of a 12-well plate with the addition of IL-2 at 300 units/mL. Afterwards, the cell growth was observed regularly with partial media replacement every two days with the addition of IL-2. The effectiveness of transduction was then evaluated after 5 days with the help of Flex-T technology.

### 2.12. In Vitro Cytotoxicity Assessment

Following 7 days after transduction, the transduced cells were collected, centrifuged at 350 g for 10 min, and the cell count and viability were assessed in a counting chamber with trypan blue staining. In parallel, tumor cells were collected in the log phase using a trypsin solution, consisting of 0.1% trypsin (PanEco, Moscow, Russia) in a 1:4 ratio with EDTA solution (Vector, Novosibirsk, Russia), and were transferred into the wells of a 96-well plate at a concentration of 5 × 10^3^ cells/well; after 2–3 h, T cells were added at a concentration of 5 × 10^4^ cells/well. Thus, the Effector to target ratio was 10:1. The cells were incubated for 16–18 h in medium with 5% FCS (LT Biotech, Vilnius, Lithuania), after which the cytotoxicity was assessed using LDH assay (J2381, Promega, Madison, WI, USA) according to the manufacturer’s instructions.

## 3. Results

### 3.1. Induction of HER2/Neu-Specific T-Lymphocyte

Upon reviewing the literature, the HER2/neu HLA-A2-binding peptide KIFGSLAFL (KIF, p369-377) was selected as the target peptide for preparing antigen-specific T cells. The HLA-A2 was chosen since it is the most frequent class I HLA genotype among almost all human populations [21]. In their turn, the donors were pre-selected for the presence of the HLA-A02 genotype based on flow cytometry screening and for the presence of KIF-specific T cells using Flex-T technology.

**Figure 2:**
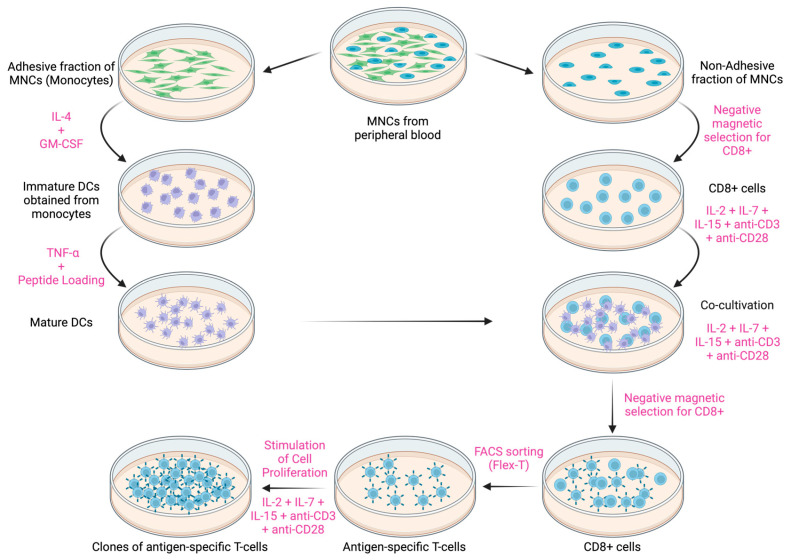
Schematic diagram of the developed protocol for the induction of antigen-specific cytotoxic T lymphocytes in vitro. PBMCs: peripheral blood mononuclear cells. DCs: Dendritic cells, created with BioRender.com.

Due to the low frequency of cells with TCRs of the needed specificity in the lymphocyte pool, a protocol was developed for the induction of preparative amounts of antigen-specific cytotoxic T lymphocytes in vitro (Figure 2).

**Figure 3:**
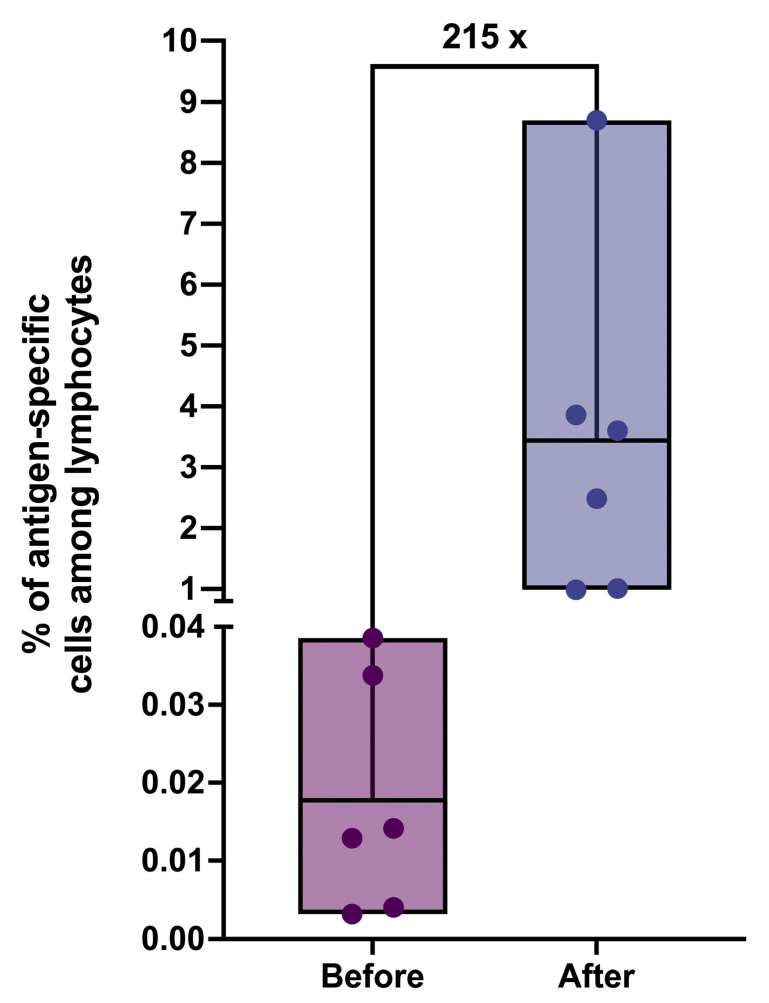
The percentage of antigen-specific T cells among lymphocytes for donors before and after applying the induction protocol (*n* = 6); the line splitting the box in two corresponds to the mean value.

With the help of the developed protocol, we were able to achieve a remarkable increase in the percentage of antigen-specific T cells among lymphocytes population by more than 200-fold (Figure 3). The average percentage of antigen-specific cells at the output varied depending on the donor and ranged between 1 and 8,7%, which is sufficient for effective sorting. The purity of the sorted cells ranged from 50 to 68%, with the total cell count ranging from 25 to 170 thousand cells.

### 3.2. Single-Cell Sequencing and Selection of TCR Clonotypes

The single-cell immune transcriptome and TCR profiling of the cultivated cells were successfully performed on the BD Rhapsody platform and gene expression, and Adaptive Immune Receptor Repertoire (AIRR) matrices were obtained for further single-cell analysis.

Of the 18,000 HER2-specific CD8 T cells that were loaded into the BD Rhapsody cartridge for the experiment, 4989 single cells (27.7%) and 1429 TCR clonotypes were successfully sequenced, analyzed, and passed the quality control (QC) measures of the BD Rhapsody pipeline. We first analyzed the TCR clonotypes using the ERGO-II (pEptide tcR matchinG predictiOn) neural network [12] and obtained predicted TCR–peptide–MHC binding scores. Using ERGO-II, we can predict the binding specificity of the obtained TCRs to the target peptide, facilitating selection between candidate TCRs based on the desired application.

We then counted clonotypes using the TCRscape tool [11] and filtered out the clonotypes that were represented by one cell only to prepare for further in-depth high-dimensional analysis, which left us with 110 TCR clonotypes (7.7%). As a result, we discovered three main clonotypes, based on the theory of clonal expansion [22], with each clonotype expressed in seven cells. From there, with the help of the predicted binding scores obtained from ERGO-II, the clonotype with the highest binding specificity among them was designated to be the dominant one. This approach combine clonal expansion of the most fit clones with binding score predictions to accurately select the candidate TCR.

**Figure 4:**
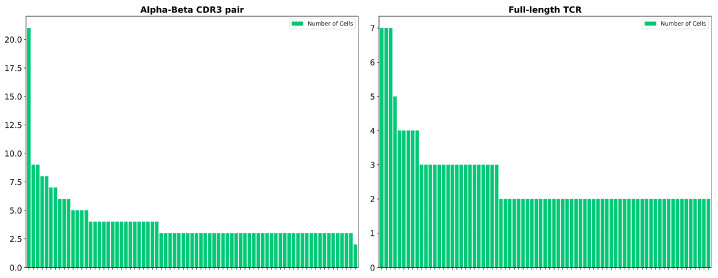
Difference between the number of cells expressing the same alpha–beta CDR3 pair compared to full-length TCR. Each bar represents a different clonotype.

It is also worth mentioning that there was a notable difference between the number of cells expressing the same alpha–beta CDR3 (Complementarity Determining Region 3) pair compared to the number of cells expressing the same TCR (Figure 4). Specifically, the dominant alpha–beta CDR3 pair was expressed in 21 cells, 7 of which are the cells that expressed the dominant TCR clonotype, while the remaining cells expressed TCR clonotypes represented by only 2 cells each. This finding supports the notion that not only the CDR3 of the beta chain but also the CDR3 regions of both alpha and beta chains are insufficient for determining TCR binding specificity, and this highlights the critical role that other parts of the TCR play in this process, such as V and J chains [12,23,24].

**Figure 5:**
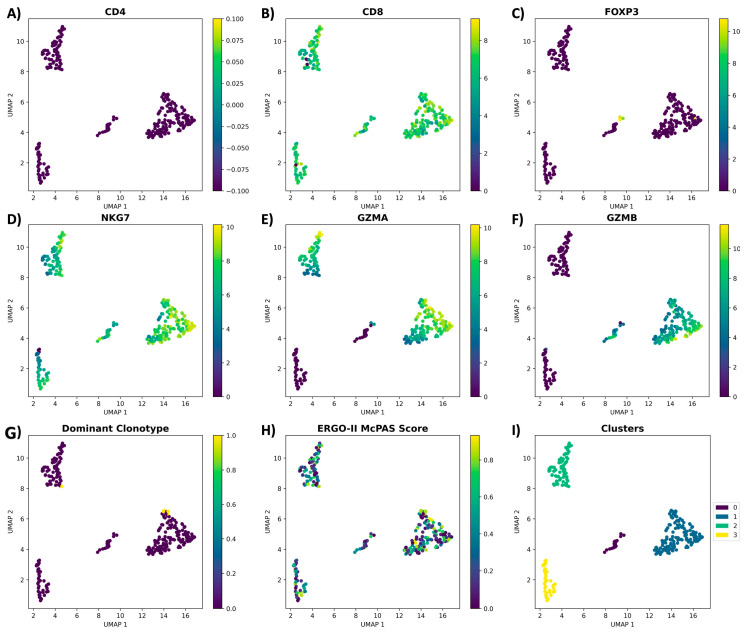
Clonotype and transcriptome analysis of the antigen-specific T cells via TCRscape tool; (**A**) *CD4*; (**B**) *CD8*; (**C**) *FOXP3*; (**D**) *NKG7*; (**E**) *GZMA*; (**F**) *GZMB*; (**G**) dominant clonotype distribution; (**H**) predicted binding specificity of clonotypes by ERGO-II; (**I**) clusters found by HDBSCAN. Yellow color at the scale represents the highest expression, and purple color represents the lowest expression.

For the in-depth high-dimensional analysis, we performed dimensionality reduction and clustered the cells based on their: clonotype, cell count per clonotype, CD4, CD8, FOXP3, NKG7, GZMB, and GZMA genes expression, as well as the ERGO-II predicted binding score. As a result, we identified four main clusters with cells expressing the dominant clonotype clustered together (with the exception of one cell) and exhibiting a cytotoxic transcriptome: CD8+ FOXP3− NKG7+ GZMA+ GZMB+ (Figure 5).

### 3.3. Plasmid Construction and MOI Identification

Based on the sequencing data and with the help of ExpOptimizer Codon Optimization Tool [20], the insert of the lentiviral transfer plasmid encoding for the dominant TCR clonotype was prepared. The insert included the TCRa and TCRb sequences in a single reading frame, separated by a signal to reset the polypeptide with the P2A polypeptide, along with the 5′ and 3′ untranslated regions (UTRs) of β-globin gene to insure efficient translation and mRNA stability [25,26] (Figure S4).

**Figure 6:**
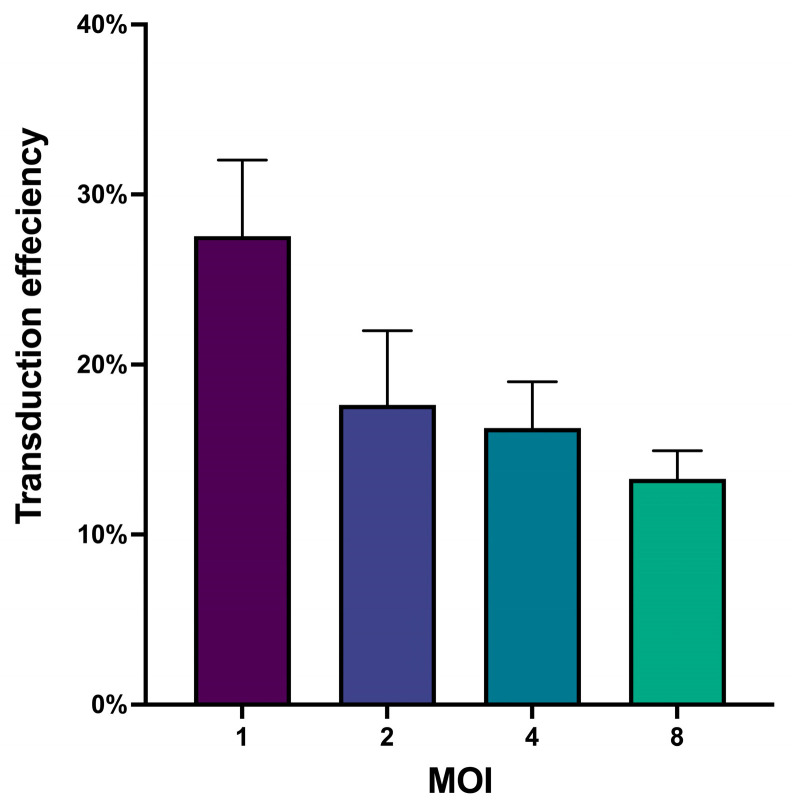
Bar plot for the transduction efficiencies across different MOI, (*n* = 4), data represented as mean with SD.

After packaging and titrating the lentivirus, it was essential to determine the optimal number of viral particles required for maximum cell transduction while maintaining high viability [27]. To achieve this, we conducted a pilot transduction experiment on CD3+ cells to identify the Multiplicity Of Infection (MOI) for them. The MOI represents the ratio of infectious agents (lentiviral transforming units (TFU)) to infection targets (target cells) [28]. Following transduction, we assessed both cell viability and transduction efficiency. All tested MOIs reported high cell viability (>95%); however, the transduction efficiency varied across different MOIs (Figure 6), highlighting the importance of titrating viruses used for transduction and optimizing the MOI for the targeted cells to achieve maximum transduction efficiency and ensure data reproducibility.

Based on the obtained results, an MOI of 1 was selected for subsequent transduction experiments.

### 3.4. In Vitro Antitumor Assessment for the Candidate TCR

To assess the ability of the candidate TCR to recognize the HER2/neu antigen and promote T cell activation, we conducted an in vitro tumor antigen stimulation experiment. This involved co-culturing T cells expressing the putative TCR with three different cancer cell lines expressing the antigen of interest at different levels: SK-MEL-5, which shows high HER2/neu expression levels (HER2/neu+++); HCT-116, which displays moderate HER2/neu expression levels (HER2/neu+); and MDA-MB-231, with minimal HER2/neu expression in a small subset of cells (HER2/neu+/−). Details on HER2/neu expression on these cell lines, verified by flow cytometry, are provided in the Supplementary Materials and Figure S1.

**Figure 7:**
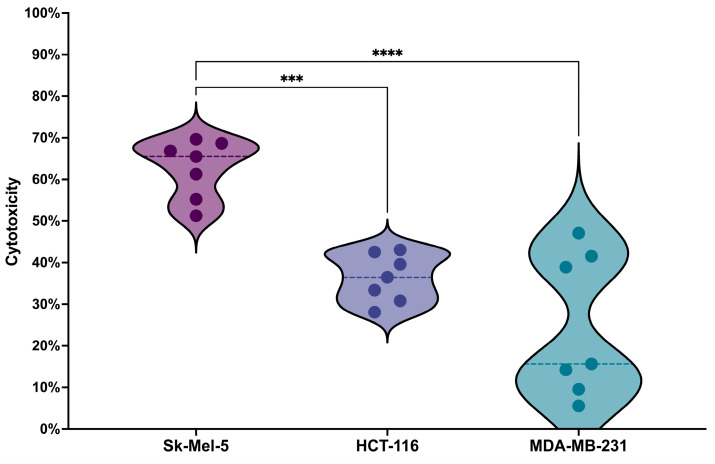
Violin plot for the cytotoxicity analysis results, representing the percentage of tumor cell death in the HER2/neu-highly expressing cell line (SK-MEL-5), the HER2/neu-moderately expressing cell line (HCT-116), and the cell line with minimal HER2/neu expression (MDA-MB-231); details on HER2/neu expression on these cell lines, verified by flow cytometry, are provided in the Supplementary Materials and Figure S1. Data are presented from 7 biological replicates, each with 4 technical replicates, the dotted line represents median values. ***: Adjusted *p* Value = 0.001. ****: Adjusted *p* Value < 0.0001.

The results revealed a statistically significant difference in cytotoxicity against the SK-MEL-5 cell line compared to HCT-116 and MDA-MB-231 (Figure 7). This result can serve as evidence for the selectivity of the candidate TCR, as it shows that T cells expressing this TCR can preferably target tumor cells characterized by a 100–200-fold increase in HER2/neu receptor concentration [9] while sparing normal cells that have lower levels of HER2/neu receptors.

## 4. Discussion

In this study, with the help of single-cell RNA sequencing, bioinformatics, and machine learning, we designed a comprehensive protocol for analyzing the repertoire of naturally occurring TCRs specific to any antigen of interest and identifying candidate TCRs potentially suitable for designing TCR-T adoptive cell therapy targeting tumors bearing the targeted antigen. The developed protocol solves the problem of sample scarcity by enriching the percentage of antigen-specific T cells by more than 200-fold in the initial population. Furthermore, our approach significantly outperformed existing approaches using multiplex PCR or 5′-RACE, which exhibit lower accuracy and sensitivity [29]. This advantage stems from employing the cutting-edge single-cell sequencing technology and TCRscape tool [11] to screen for antigen-specific TCRs that naturally develop in the immune system, based on the clonal expansion of the most fit clones in the repertoire, taking full advantage of all the natural systems and selection processes governing TCR development. Additionally, the developed protocol makes it possible to analyze the entire repertoire of naturally-occurring antigen-specific TCRs, unlike other approaches that identify only a few [30,31], which makes our approach much more practical. Moreover, our protocol harnesses the powerful capabilities of machine learning algorithms via the use of ERGO-II [12] to predict the binding specificity of the obtained TCRs to the MHC/peptide complex, which helps fine-tune the developed adoptive therapy for tumors and allows for broader application of the analyzed TCR repertoire based on the desired application. On top of that, this protocol can be tailored to accommodate different types of antigens and MHC variants, making it a highly versatile tool for both research and clinical applications.

In the case at hand, the developed protocol was applied for the identification of candidate TCRs against the tumor-associated antigen HER2/neu. Accordingly, we successfully generated preparative amounts of HER2/neu-specific T cells from the PBMCs of conditionally healthy donors via enriching the percentage of antigen-specific T cells by more than 200-fold. Subsequently, we conducted single-cell sequencing for the immune transcriptome and TCR repertoire of the prepared cells, which resulted in the identification of more than 100 TCR clonotypes, followed by further in-depth analysis of the clonotypes’ count, transcriptome, and binding specificity to the MHC/peptide complex, as a result of which a dominant clonotype with medium binding specificity to the targeted HER2/neu peptide was discovered. From there, replication-incompetent lentiviruses carrying the gene encoding for the candidate TCR were obtained and titrated for the preparation of TCR-T cells against HER2/neu-bearing tumors. Furthermore, the antitumor selective cytotoxicity of the candidate TCR was verified, indicating its potential as a suitable candidate for designing TCR-T adoptive cell therapy against HER2/neu-bearing tumors.

Furthermore, the study revealed a significant disparity between the number of cells expressing identical alpha–beta CDR3 pairs and cells with identical full-length TCRs. This discrepancy emphasizes the fact that not only the beta chain’s CDR3 alone is insufficient for determining TCR binding specificity but also that the CDR3 regions of both alpha and beta chains collectively are insufficient for this, underscoring the essential role played by other components of the TCR, such as the V and J chains, in determining TCR binding specificity [12,23,24].

## 5. Patents

The clonotype investigated in this article is currently being patented with the Patent of the Russian Federation with Application Number 2023133340.

## 6. Conclusions

This study presents a robust and versatile protocol for identifying naturally occurring TCRs specific to any antigen of interest, offering significant advancements in TCR discovery and adoptive cell therapy design. By integrating single-cell RNA sequencing, bioinformatics, and machine learning tools, we developed a protocol that not only enriches antigen-specific T cells by over 200-fold but also outperforms traditional approaches in accuracy and sensitivity. The protocol’s ability to analyze the entire repertoire of antigen-specific TCRs, coupled with its flexibility to accommodate various antigens and MHC variants, makes it a powerful tool for both research and clinical applications.

Applying the protocol to HER2/neu, a tumor-associated antigen, enabled the identification of a dominant TCR clonotype with potential therapeutic utility. The functionality of this candidate TCR was demonstrated through the generation of HER2/neu-specific TCR-T cells and their selective cytotoxicity against antigen-bearing tumors. Additionally, our findings underscore the complexity of TCR binding specificity, highlighting the critical contribution of both the alpha and beta chains.

Overall, the developed protocol paves the way for precise, efficient, and customizable TCR-T cell therapies, addressing current limitations and opening new avenues for cancer immunotherapy.

## Data Availability

Single-cell gene expression data were deposited to Zenodo: https://zenodo.org/doi/10.5281/zenodo.13120535, accessed on 29 July 2024.

## Supplementary Materials

The following supporting information can be downloaded at https://www.mdpi.com/article/10.3390/cancers16234020/s1: Figure S1: The Percentage of HER2/neu-positive cells and the corresponding Mean Fluorescence Intensity (MFI) values across various cell lines.; Figure S2: The gating strategy for isolating antigen-specific T cells from lymphocytes population using Flex-T technology.; Figure S3: Full sequence map for the pLenti hPGK GFP lentiviral vector.; Figure S4: Full sequence map for the prepared lentiviral transfer plasmid insert.
