# Supplementary material for: Targeting Precision in Cancer Immunotherapy: Naturally-Occurring Antigen-Specific TCR Discovery with Single-Cell Sequencing
Source: Cancers (Basel). 2024 Nov 30;16(23):4020. doi: 10.3390/cancers16234020 (PMC11640272; doi:10.3390/cancers16234020)
Supplement: Supplementary file 1 [file cancers-16-04020-s001.zip › cancers-3248190-supplementary.pdf]

Supplementary Materials

Targeting Precision in Cancer Immunotherapy: Naturally-Occurring Antigen-Specific TCR Discovery with Single-Cell Sequencing

Saleh Alrhmoun[1,2], Marina Fisher[1], Julia Lopatnikova[1], Olga Perik-Zavodskaia[1], Marina Volynets[1], Roman Perik-Zavodskii[1], Julia Shevchenko[1], Kirill Nazarov[1], Julia Philippova[1], Alaa Alsalloum[1], Vasily Kurilin[1], Alexander Silkov[1], and Sergey Sennikov[1,3,*]

[1] Laboratory of Molecular Immunology, Research Institute of Fundamental and Clinical Immunology; Novosibirsk, Russia.

[2] Department of Natural Sciences, Novosibirsk State University; Novosibirsk, Russia.

[3] Institute of Medicine and Medical Technologies, Department of Immunology; Novosibirsk State University,630090 Novosibirsk, Russia.

[*] Correspondence: E-mail: sennikov@niikim.ru

Supplementary materials and methods

PBMCs Isolation

Peripheral blood was collected into vacuum tubes with EDTA anticoagulant and the peripheral blood mononuclear cells (PBMCs) were isolated using a conventional Ficoll–Urografin density gradient method. Briefly, the peripheral blood was diluted onefold with RPMI-1640 medium (1.3.4., Biolot, Russia) and was then carefully layered on top of a Ficoll–Urografin solution (ϱ = 1.077 g/L, 17-1440-03, PanEco, Russia) and subjected to centrifugation at 400 g and room temperature for 40 min. Subsequently, the mononuclear cells were collected from the buffy coat located at the phase boundary, spanning the entire cross-section of the tube.

Lentivirus Preparation

The Human embryonic kidney 293T (HEK293T) packaging cells were used for the production of lentiviruses, the HEK293T cell line was generously provided by Dr. Hiroshi Shiku (Mie University, Japan). After thawing, the cells were cultured in T75 flasks (TRP, Switzerland) at an initial count of 1×10^6 cells in 25 ml full DMEM medium (PanEco, Russia). The medium was supplemented with HEPES 10 nmol/m, glutamine 600 ng/ml (BioloT, Russia), gentamicin 10 ng/ml (PanEco, Russia), benzylpenicillin

200 units/ml (BioloT, Russia), 2-mercaptoethanol 350 pmol/ml (Sigma, USA) and FCS 10% (v/v) (LT Biotech, Lithuania), the supplements are indicated as final concentrations. 1 passage after thawing, when the cells reached ~ 70-90% confluence, they were considered ready for transfection and were detached from the flask surface by 5-7 minutes incubation at 37 °C in 10 ml of trypsin solution consisting of 0.1% trypsin (PanEco, Russia) in a 1:4 ratio with EDTA solution (Vector, Russia). The detachment process was monitored under the microscope and the detached cells were then collected into a 50 ml falcon test tube, while the flask was washed a couple of times with 20 ml of PBS, collecting everything in the 50 ml test tube. The cells were then centrifuged at 300 g for 8 min followed by carefully draining the supernatant with the subsequent resuspension of the cell pellet in 1 ml of PBS. After that, the cell count and viability were assessed in a counting chamber with trypan blue staining, ensuring that only cells with a viability exceeding 90% were used for transfection.

2×10^6 cells were then transferred into a 1.5 ml Eppendorf in preparation for transfection. In parallel, 150 μl of Opti-MEM medium (Gibco, USA) and 8 μl of Lipofectamine 2000 (Thermo Fisher) were combined in one Eppendorf tube, while in another, 150 μl of Opti-MEM medium and 10 μg of plasmids were combined, 2.5 μg of each plasmid (Gag-pol, Rev, VSV-G encoding plasmid and the Transfer plasmid). The plasmid mixture was then combined with the Lipofectamine 2000 solution in the first tube and the mixture was incubated for 5 minutes at room temperature. Following that, the mixture was added to the cell suspension in PBS, gently mixed and allowed to further incubate for 5 minutes at room temperature. Next, the cells were transferred to a 100 mm diameter Petri dish containing 12 ml of DMEM medium supplemented with HEPES, glutamine, 2-mercaptoethanol. Antibiotics and FCS are not used here as they can inhibit lipofectamine. The cells were allowed to grow for 3 days at 37 °C and 5% CO2, after which, the supernatant containing lentivirus was transferred into a test tube and centrifuged at 300 g for 8 minutes to precipitate the cells. After centrifugation, the supernatant, now free of cells, was carefully transferred to another tube containing TransLv™ Lentivirus Precipitation Solution (5×) (TransGen,

China), with a 4:1 volume ratio of supernatant:precipitation solution. The resulting mixture was then gently mixed and incubated at +4 °C overnight. The following day, the mixture was centrifuged at 1500 g for 45 min with cooling to + 4 °C. After centrifugation, the supernatant was carefully drained or pipetted off leaving a small white precipitate at the bottom of the test tube that contained the virus. Finally, the virus sediment is resuspended in 0.5 ml of cold PBS and transferred to a cryoprobe that was kept at -150 °C until later use.

Lentivirus titration

The obtained lentiviruses were titrated using TransLv™ Lentivirus qPCR Titration Kit (TransGen, China) according to the manufacturer's instructions. For that, the HEK293T cells were selected as target cells due to their high susceptibility to transduction.

HEK293T cells were thawed and prepared as described above. After cell count and viability check, the cells were transferred to a 12-well plate (TRP, Switzerland) at a concentration of 1×10^5 cells/well. Then 100 µl of the obtained lentivirus were added to each well, with the total volume adjusted to 1 ml using DMEM medium without FCS and antibiotics. The cells were then incubated for one day at 37 °C and 5% CO2, after which, the plate was centrifuged at 250 g for 8 minutes to precipitate the cells, followed by carefully collecting all the medium from the wells and replacing it with 1 ml of fresh DMEM medium without FCS and antibiotics. The cells were then allowed to further incubate for another day. After 48 h from the beginning of transduction, the medium was carefully aspirated from the wells and the cells were collected as described above. The cells were then centrifuged at 300 g for 8 min and the cell pellet was subsequently used for isolating the DNA with the help of Total Genomic DNA Isolation Kits (Norgen Biotek, Canada) according to the manufacturer's instructions. The DNA concentration was assessed by Qubit 4 with the High-Sensitivity dsDNA Kit (Thermo Fisher, USA) and the lentiviral titer was calculated using TransLv™ Lentivirus qPCR Titration Kit.

HER2/neu expression on tumor cell lines

10^6 cells from each cell line were washed in PBS containing 0.09% NaN_3 and stained for the expression of HER2/neu with the BioLegend (San Diego, California, United States) antibody #324406 PE anti-human CD340 (erbB2/HER-2) Antibody according to the manufacturer's instructions. We then washed the cells after 30 minutes of incubation in the dark with 0,5 ml PBS containing 0.09% NaN_3 and subsequently analyzed using an Attune NxT flow cytometer (Thermo Fisher,USA). We then manually gated cells from debris, and HER2/neu-positive cells from cells using unstained control for each cell line. After that the Percentage of HER2/neu-positive cells and the Mean Fluorescence Intensity (MFI) were exported to GraphPad Prism 9.4 where they were plotted (Figure S1).

Supplementary figures

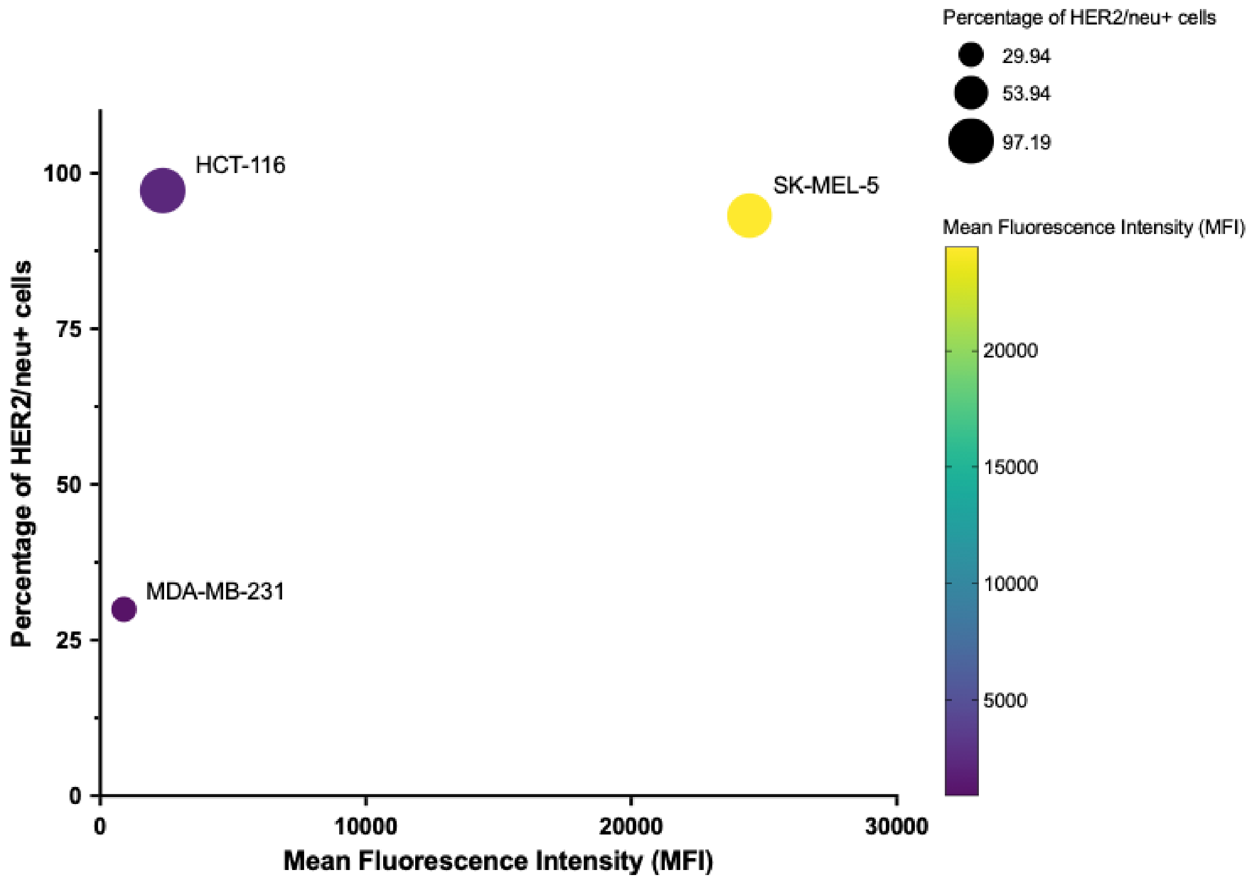

Figure S1. The Percentage of HER2/neu-positive cells and the corresponding Mean Fluorescence Intensity (MFI) values across various cell lines.

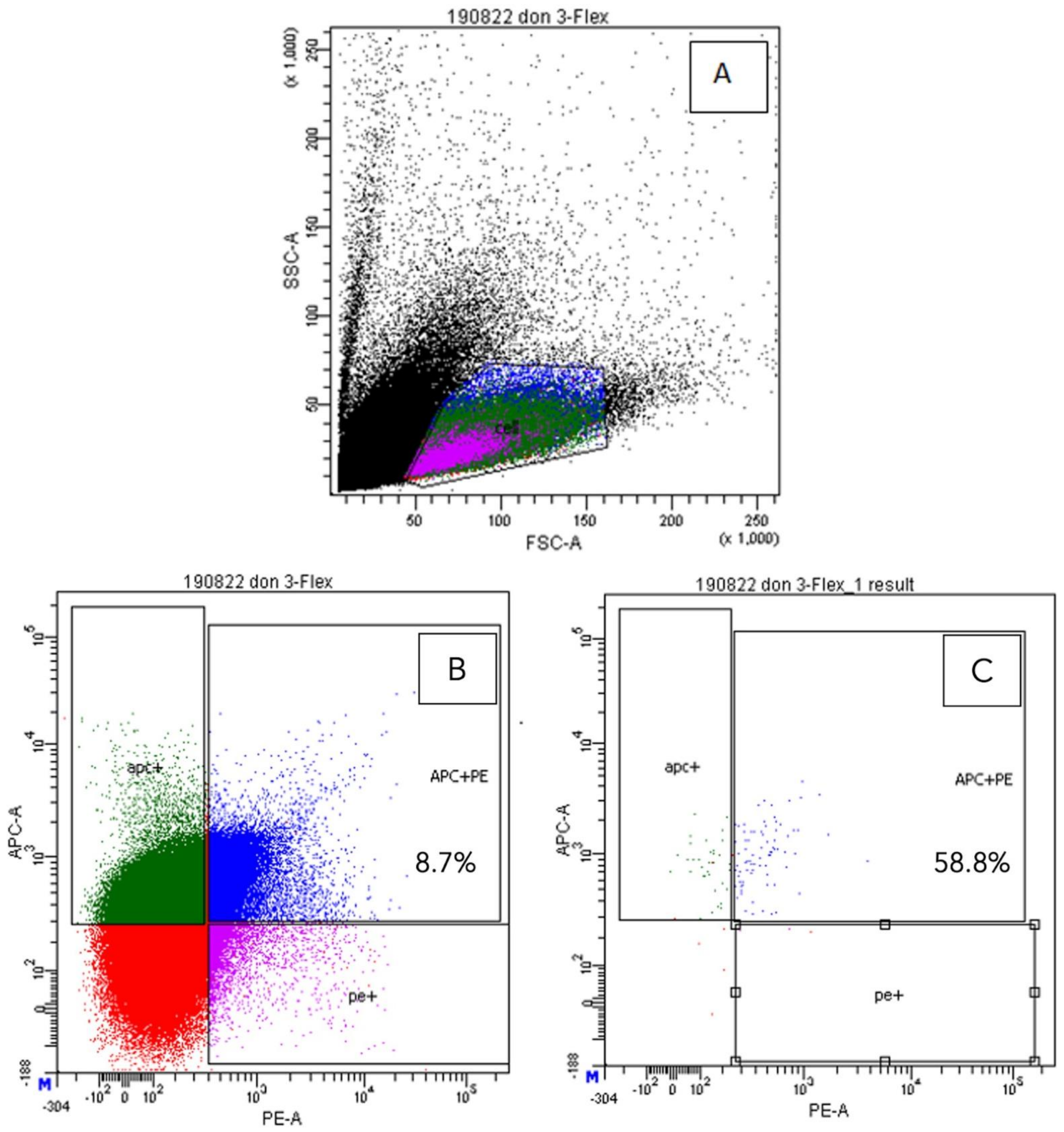

Figure S2. The gating strategy for isolating antigen-specific T cells from lymphocytes population using Flex-T technology. A) Cell distribution by forward and side scatters to gate the morphologically viable lymphocytes; B) The antigen-specific T cell population before sorting, with the targeted cells stained simultaneously by tetramers coupled to either APC or PE fluorochromes; C) The antigen-specific T cell population after sorting. The percentages shown represent double-positive cells.

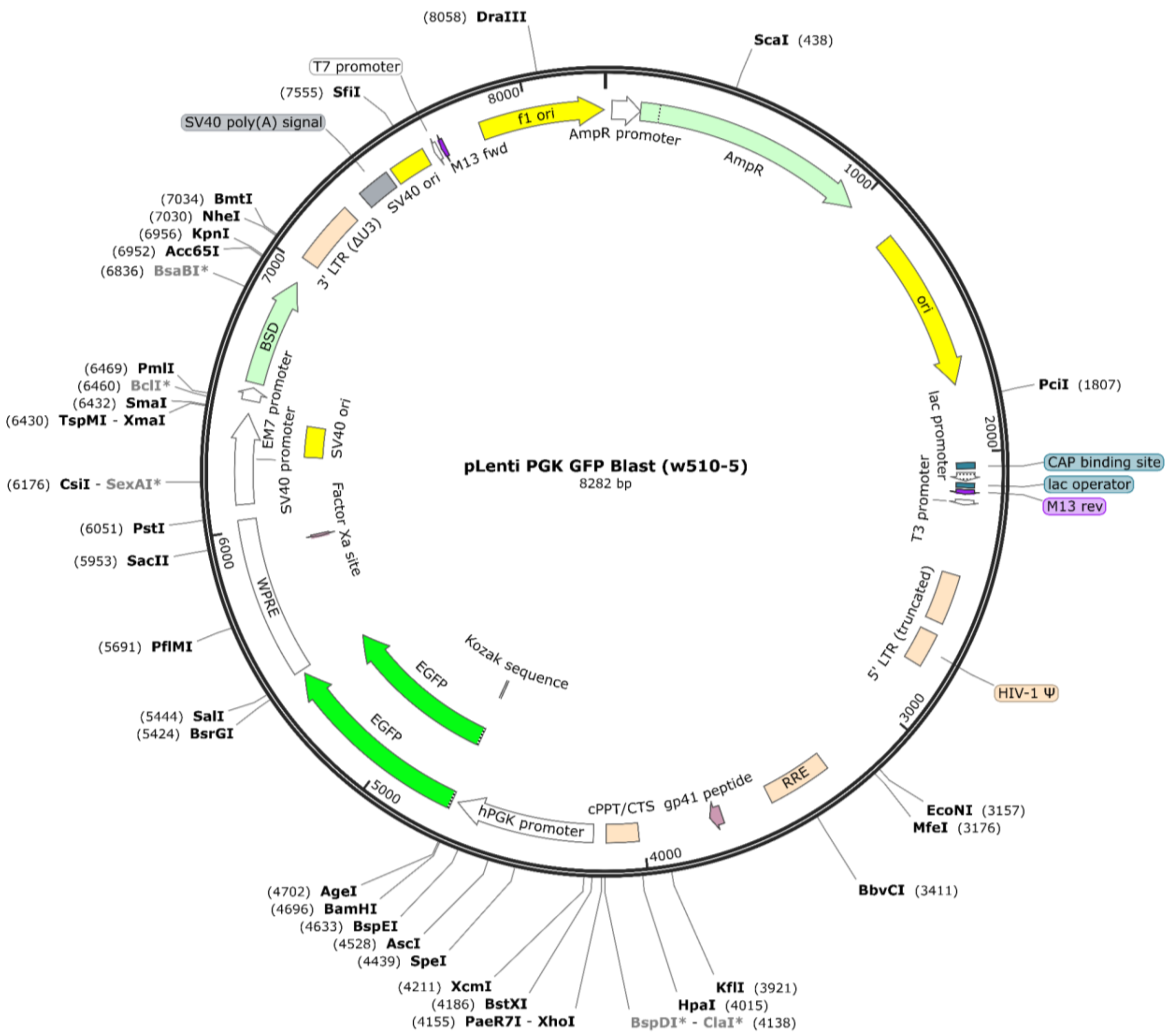

Figure S3. Full sequence map for the pLenti hPGK GFP lentiviral vector.

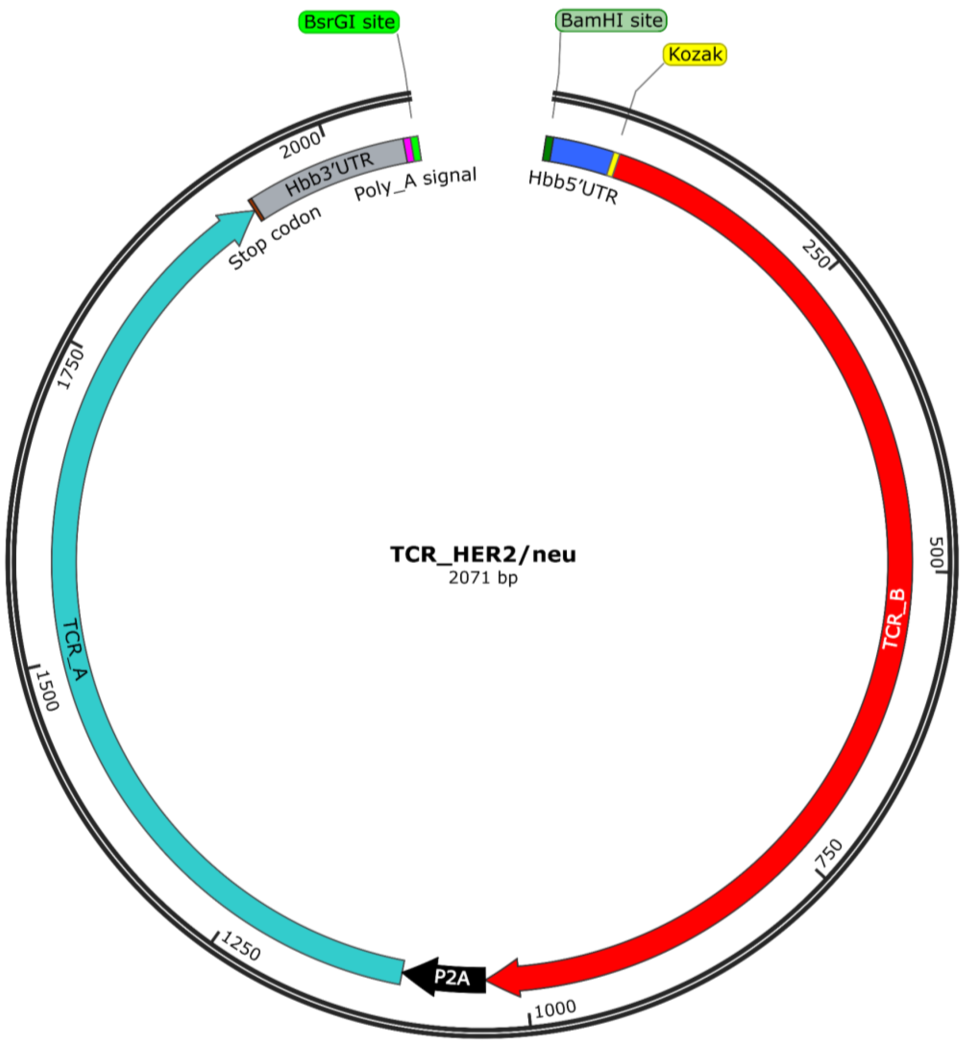

Figure S4. Full sequence map for the prepared lentiviral transfer plasmid insert.
